# Sclerostin-Mediated Impaired Osteogenesis by Fibroblast-Like Synoviocytes in the Particle-Induced Osteolysis Model

**DOI:** 10.3389/fmolb.2021.666295

**Published:** 2021-06-23

**Authors:** Supriya Jagga, Ashish Ranjan Sharma, Yeon Hee Lee, Ju-Suk Nam, Sang-Soo Lee

**Affiliations:** Institute for Skeletal Aging and Orthopedic Surgery, Hallym University-Chuncheon Sacred Heart Hospital, Chuncheon, South Korea

**Keywords:** fibroblast-like synoviocyte, wear debris, osteolysis, sclerostin, bone signaling, osteoblasts, osteoclasts

## Abstract

Engineered biomaterials are envisioned to replace, augment, or interact with living tissues for improving the functional deformities associated with end-stage joint pathologies. Unfortunately, wear debris from implant interfaces is the major factor leading to periprosthetic osteolysis. Fibroblast-like synoviocytes (FLSs) populate the intimal lining of the synovium and are in direct contact with wear debris. This study aimed to elucidate the effect of Ti particles as wear debris on human FLSs and the mechanism by which they might participate in the bone remodeling process during periprosthetic osteolysis. FLSs were isolated from synovial tissue from patients, and the condition medium (CM) was collected after treating FLSs with sterilized Ti particles. The effect of CM was analyzed for the induction of osteoclastogenesis or any effect on osteogenesis and signaling pathways. The results demonstrated that Ti particles could induce activation of the NFκB signaling pathway and induction of COX-2 and inflammatory cytokines in FLSs. The amount of Rankl in the conditioned medium collected from Ti particle–stimulated FLSs (Ti CM) showed the ability to stimulate osteoclast formation. The Ti CM also suppressed the osteogenic initial and terminal differentiation markers for osteoprogenitors, such as alkaline phosphate activity, matrix mineralization, collagen synthesis, and expression levels of Osterix, Runx2, collagen 1α, and bone sialoprotein. Inhibition of the WNT and BMP signaling pathways was observed in osteoprogenitors after the treatment with the Ti CM. In the presence of the Ti CM, exogenous stimulation by WNT and BMP signaling pathways failed to stimulate osteogenic activity in osteoprogenitors. Induced expression of sclerostin (SOST: an antagonist of WNT and BMP signaling) in Ti particle–treated FLSs and secretion of SOST in the Ti CM were detected. Neutralization of SOST in the Ti CM partially restored the suppressed WNT and BMP signaling activity as well as the osteogenic activity in osteoprogenitors. Our results reveal that wear debris–stimulated FLSs might affect bone loss by not only stimulating osteoclastogenesis but also suppressing the bone-forming ability of osteoprogenitors. In the clinical setting, targeting FLSs for the secretion of antagonists like SOST might be a novel therapeutic approach for preventing bone loss during inflammatory osteolysis.

## Introduction

Artificial joint replacements are a remarkably effective and safe method for treating patients with degenerative diseases such as osteoarthritis (OA) and inflammatory arthropathies, including rheumatoid arthritis (RA) (Mulhall et al., 2008; Shon et al., 2019). Each year, several million people (>1.3 million) undergo total joint arthroplasty (TJA) worldwide (Veronesi et al., 2017). Wearing of prosthetic implants during the progression of time is the major problem, necessitating revisions after TJAs (Fraser et al., 2015). The term “periprosthetic osteolysis” is often referred to as a progressive insidious bone resorption event associated with a well-functioning total hip arthroplasty (THA). In most cases, periprosthetic osteolysis is usually followed by aseptic loosening, which can be described as a failure of the implant due to poor initial fixation and mechanical damage to fixation over time or biological loss of fixation instigated by immunological reactions to wear debris particles (Gilbert et al., 2016).

Both biological and mechanical factors might be the contributing factors to the pathogenesis of implant loosening (Wilkinson et al., 2003; Wilkinson et al., 2005). Even genetic variations among the inflammatory and bone turnover signaling pathways are crucial factors for assessing patients’ susceptibility to osteolysis (Jagga et al., 2019). Though pharmacological interventions are being considered for treating osteolysis (anti–bone resorptive drugs like bisphosphonates), most of them are effective only once osteolysis is induced. Usually, it is observed that 2–3 months after surgery, a fibrous membrane, irregularly organized (abundant in fibroblasts, macrophages, chondrocytes, lymphocytes, endothelial cells, mesenchymal stem cells (MSCs), and prosthesis-derived wear particles) and resembling more or less synovial tissue, may form around the bone/prosthesis interface (Zhang et al., 2010). Most of these cell types are able to phagocyte the wear particles and get activated. The activation of monocyte/macrophage cell lineage is regarded as the initial stage of the body’s biological reaction to wear debris generation. An elevated secretion of various kinds of pro-inflammatory cytokines and mediators such as interleukin (IL)-6,-8,-11, and -1β, tumor necrosis factor (TNF)-α, transforming growth factor (TGF)-α and -β, and prostaglandin E2 (PGE2), chemokines such as monocyte chemoattractant protein 1 (MCP-1) and matrix metalloproteinases (MMPs), and osteoclast-promoting factors such as receptor activator of nuclear factor kappa-Β ligand (Rankl) has been observed (Purdue et al., 2006; Murray and Wynn, 2011; Gallo et al., 2013). The role of released inflammatory mediators as well as osteoclastogenic factors by various immune cells in enhancing the osteoclastic activity after the generation of wear debris has been well studied. However, the factors that might contribute to suppressed bone formation and bone loss as observed during periprosthetic osteolysis remain elusive. It has recently been demonstrated that cytokine-like TNF-α from Ti particle–stimulated macrophages suppresses osteoblasts’ osteogenic activity (Lee et al., 2012). Moreover, the direct exposure to wear debris affects osteoblasts’ bone-forming ability and stimulates them to release bone-resorbing agents such as Rankl and macrophage-colony stimulating factor (M-CSF) (Nam et al., 2017b). However, there are still very few investigations that have addressed the possible role of other cell types in bone loss during periprosthetic osteolysis.

Tissue-resident fibroblast-like synoviocytes (FLSs) constitute about 70% of the cellular population in the periprosthetic membrane and are in direct contact with wear debris from the prosthesis (Perry et al., 1995). Under inflammatory conditions like rheumatoid arthritis, FLSs have been shown to secrete inflammatory mediators and matrix-degrading proteases (Pap et al., 2000). Recently, the importance of FLSs has been acknowledged in the pathogenesis of aseptic loosening following TJAs (Broeren et al., 2019). By controlling the composition of the synovial fluid and the extracellular matrix, FLSs maintain the dynamic integrity of joints. FLSs present in the interface membranes of patients with failed TJAs have been shown to respond to wear debris with elevated expression of bone-resorbing MMPs, stromelysin, and collagenase, leading to the destruction of the extracellular matrix of bone (Cao et al., 2013). Moreover, once stimulated with wear debris, FLSs have been attributed to the behavior of releasing pro-inflammatory cytokines such as TNF-α, IL-6, IL-1β, IL-8, MCP-1, and Rankl, contributing to the enhancement of osteoclastogenesis (Green et al., 1998; Sharma et al., 2020). Lately, it has been shown that Ti particles from articulate surfaces of the implant can activate FLSs in the direction of inflammation that might contribute in an autocrine and paracrine manner to create a complex milieu within the periprosthetic space. To date, any role or contribution of FLSs in affecting bone loss during periprosthetic osteolysis is still mysterious. Thus, the objective of the current study was to understand the effect of Ti particles on human FLSs and the mechanism by which they might affect or participate in the process of bone loss during periprosthetic osteolysis.

## Materials and Methods

### Preparation of Ti Particles

Ti particles were commercially procured from Johnson Matthey Company (Ward Hill, MA). As per the manufacturer datasheet, histological analysis revealed that the Ti particles had an average diameter of 5.34 μm, and 90% of particles were <10.0 μm in size. Endotoxin-free Ti particles were prepared as described previously (Nam et al., 2017b). Ti particles were sonicated and vortexed before each treatment.

### Cell Culture

Primary FLSs were harvested from human synovial tissue obtained during total knee replacement surgery at Hallym University Sacred Heart Hospital. The ethical committee of Hallym University Sacred Heart Hospital approved and permitted the experimental protocol. All contributors signed a written informed consent (number: 2009-42). Primary FLSs were isolated as per the previously described protocol with some modifications (Rosengren et al., 2007). In brief, the freshly obtained biopsied tissue section of 5 µm was thoroughly washed with PBS before and between its sequential digestions. The tissue was incubated twice at 37°C in the water bath with serum-free Dulbecco’s modified Eagle’s medium (DMEM: Invitrogen, Carlsbad, CA) having 1 mg/mL collagenase type IV (Sigma Aldrich) and 10% penicillin/streptomycin (P/S) (Lonza, Walkersville, United States) at concentrations of 100 IU/mL (*p*) and 100 μg/mL (S) for 60 min. After the second enzymatic dispersion, tissues were strained to separate the cells. Harvested cells were suspended into fresh media and were allowed to adhere to the tissue culture dishes’ surface at 37 °C temperature and 5% CO_2_ humidified atmosphere. On the second day of culture, only two major cell populations were left after the removal of non-adherent cells (CD68, expressing macrophage-like synoviocytes or fibroblast-like synoviocytes, expressing prolyl 4-hydroxylase, vimentin, and CD248). Further passage or trypsinization process resulted in a relatively homogenous population of dominant cell type, FLSs, and the removal of macrophage-like synoviocytes. The cells were kept in an incubator maintaining a 5% CO_2_ humidified atmosphere and were passed between every 3–4 days. Cells harvested from the synovial tissues were visualized microscopically, and the passage above four to six was utilized for experimental purposes. FLSs showed a typical spindle-shaped fibroblastic phenotype that formed parallel clusters at confluence (Supplementary Figure S1A). For characterization, the mRNA expression of FLSs, osteoblasts, and monocyte/macrophage-associated markers was assessed by RT-PCR (Supplementary Figure S1B–H). The majority of isolated cells expressed FLS-specific markers confirming the homogeneity of isolated FLSs.

During hip replacement surgeries of healthy patients (58- to 80-year-olds), primary human osteoblasts (HOBs) were harvested from proximal femur specimens. The Hallym University Sacred Heart Hospital (number; 2009–41) ethics committee authorized and granted permission for the followed experimental protocol. Cancellous bone fragments were cultured as per our well-established lab protocol (Lee et al., 2012).

The THP-1 human monocytic cell line (ATCC, United States) was cultured and maintained in the RPMI-1640 growth medium having 10% FBS, l-glutamine (2 mM), and 1% P/S in a humidified 5% CO_2_ atmospheric incubator at 37°C. Before the experiments, the cells were grown on a six-well plate, and 20 nM phorbol 12-myristate 13-acetate (PMA) was added for 48 h to induce differentiation into macrophage-like cells.

The human osteoblast cell line (SaOS-2: ATCC, HTB-85) and murine macrophage/monocyte cell line, RAW 264.7 cells (ATCC, TIB-71), were cultured in complete DMEM having 10% FBS, 100 °U/mL penicillin, and 100 U/mL streptomycin supplements in a humidified incubator with 5% CO_2_ and 37°C temperature.

### Differentiation of Osteoblasts

SaOS-2 cells were seeded in six-well plates at a confluence of about 1 × 10^5^ cells per well containing 1 mL media. The cells were maintained in an incubator having 37°C temperature, 95% of the humidified atmosphere, and 5% CO_2_. To induce osteoblast differentiation, osteoblast cells were grown in a specified osteogenic medium containing differentiation-inducing agents, ascorbic acid (50 μg/mL), and β-glycerophosphate (10 mM) (Sigma Aldrich), for 7 days.

### MTT Assay

The MTT, 3-(4,5-dimethylthiazol-2-yl)-2,5-diphenyl-tetrazolium bromide (Sigma Aldrich), reduction assay was performed to assess the viability of the cells according to our lab protocol (Nam et al., 2017b). 5 mg/mL of MTT reagent was added to the cells in the 96-well plate and incubated at 37 °C for 3–4 h. After discarding the supernatant, 200 µL of dimethyl sulfoxide (DMSO) was added in each well to dissolve visible insoluble formazan crystals of purple color. Finally, the optical density was determined at a wavelength of 570 nm using a plate-reading spectrophotometer.

### Lactate Dehydrogenase (LDH) Activity Assay

A cytotoxicity detection kit (Roche Diagnostics, Indianapolis, IN, United States) was used to analyze the LDH activity as per instructions of the manufacturer’s protocol with some modification (Nam et al., 2017b). To assess the cell cytotoxicity, 10 μL of cell culture media from the experimental sample was added into a new 96-well plate containing PBS (40 μL). Afterward, 50 μL of the prepared LDH reagent with its catalyst was added to each well for incubation of 45 min. The reduction of the tetrazolium salt to formazan was observed at 490 nm wavelength. For positive control of cell death, complete cell lysate was utilized.

### Collection of Conditioned Media From Ti Particle–Stimulated FLSs (Ti CM)

FLSs were seeded at a confluence of 4.0 × 10^5^ cells/60 mm tissue culture dish in complete DMEM having 10% FBS and 1% P/S overnight. After that, the cells were treated with Ti particles in serum-free DMEM containing 1% P/S for 48 h. The conditioned medium (CM) was collected in sterile conditions. To eliminate the cell debris, if any, the collected CM was centrifuged and used for further experiments with SaOS-2 and RAW 264.7 cells.

### RNA Isolation and Real-Time PCR

Total RNA was collected from cells by Trizol reagent (Invitrogen). The quality and integrity of RNA were determined by evaluating the absorbance ratio (260/280) and separating the RNA on an agarose gel, respectively. First-strand cDNA was synthesized from total RNA (2 µg) with SuperScript ІІ (Invitrogen), and RT-PCR was performed according to lab manuals (Nam et al., 2017b). The thermal cycle reaction involved 50 cycles of amplification for 20 s at 95°C, 20 s at 60°C, and 25 s at 72°C. The relative mRNA expression of desired genes was normalized to the expression level of GAPDH and quantified by the double delta CT (ΔΔCT) method. The sequences of the PCR primers are recorded in Table 1.

**TABLE 1:** Primers for real-time PCR.

Target	Forward primer (5′–>3′)	Reverse primer (3′–>5′)
***Human***		
DKK1	TCA​GAC​TGT​GCC​TCA​GGA​TTG​TGT	TCT​GTA​TCC​GGC​AAG​ACA​GAC​CTT
DKK2	TGA​TGC​GGG​CCT​CCT​GAT​CAA​TTA	ACT​GGA​AGC​AAT​CAA​ATG​CGA​GGC
DKK3	TTG​GGA​GAG​TCA​GGC​AGG​GTT​AAA	TGT​CTG​CCA​ACT​GGT​AGA​GGC​AAA
DKK4	TGG​GAC​ACT​CTG​TGT​GAA​CGA​TGT	TCT​TGT​CCC​TTC​CTG​CCT​TGT​GAT
sFRP1	AGA​GCT​GCA​CTA​TCA​CGA​GCC​TTT	AGA​CCA​ATG​ACC​AGG​CCA​ATC​AGT
sFRP2	TAG​GTG​CAA​CTG​TGA​CTT​GGG​TCT	CCA​CAA​GTT​TGG​GCC​ACA​GAG​AAA
sFRP3	CCT​GCA​AAC​TGG​CCT​GCA​CTT​TAT	AGC​ATC​ATT​TGT​TCA​CCA​CAG​CCC
sFRP4	AGG​TCA​CAA​CGG​TGG​TGG​ATG​TAA	ATC​ATC​CTT​GAG​CGC​CAC​TCG​TAA
sFRP5	TGA​GGC​GGA​GGT​TTC​AGA​GTA​GAA	AGG​CAC​TGA​GAC​CCT​AAC​TCC​TTT
SOST	TTC​AGT​GCC​AAG​GTC​ACT​TCC​AGA	TTC​TTC​CAG​GAG​TTT​GTC​AGC​CGT
Noggin	CAAGAAGCAGCGCCTAAG	GTA​CTG​GAT​GGG​AAT​CCA​G
Follistatin	AGA​GCC​TGC​TTC​CTC​TGA​G	AGC​TGT​AGT​CCT​GGT​CTT​C
Chordin	CGC​ATC​AGT​GGA​CAC​ATT​G	TTC​TGC​AGC​AGC​ATA​TGA​GC
Gremlin	ATG​TGA​CGG​AGC​GCA​AAT​AC	TGG​ATA​TGC​AAC​GAC​ACT​GC
Rankl	CGT​TGG​ATC​ACA​GCA​CAT​CAG	GCT​CCT​CTT​GGC​CAG​ATC​TAA​C
Osteocalcin	GCCTTTGTGTCCAAGC	GGACCCCACATCCATAG
Runx2	ACTGGGCCCTTTTTCAGA	GCGGAAGCATTCTGGAA
Osterix	CAA​AGC​AGG​CAC​AAA​GAA​GCC​GTA	AGG​TGA​AAG​GAG​CCC​ATT​AGT​GCT
BSP	AGT​ACC​AAC​AGC​ACA​GAG​GCA​GAA	CTG​CAT​TGG​CTC​CAG​TGA​CAC​TTT
Prolyl 4-hydroxylase	TCA​AGG​TGC​TTG​TTG​GGA​AG	AAT​GGG​AGC​CAA​CTG​TTT​GC
Vimentin	TCT​CAG​CAT​CAC​GAT​GAC​CTT​G	TTG​CGC​TCC​TGA​AAA​ACT​GC
CD68	AAG​AGC​CAC​AAA​ACC​ACC​AC	AAC​TGT​GAC​GTT​TCC​ATG​GC
Osteoprotegerin	GCAGCGGCACATTGGAC	CCC​GGT​AAG​CTT​TCC​ATC​AA
CD248	TTT​TTG​GTG​GTC​CTG​CTT​GC	AGT​CAG​TGA​TGC​GCT​TGT​TG
COX-2	CCA​AAT​CCT​TGC​TGT​TCC​CAC​CCA​T	GTG​CAC​TGT​GTT​TGG​AGT​GGG​TTT
TNF-α	AAG​GAC​GAA​CAT​CCA​ACC​TTC​CCA​A	TTT​GAG​CCA​GAA​GAG​GTT​GAG​GGT
IL-1β	AAC​CAG​GCT​GCT​CTG​GGA​TTC​TCT​T	ATT​TCA​CTG​GCG​AGC​TCA​GGT​ACT
IL-8	AAG​AAA​CCA​CCG​GAA​GGA​ACC​ATC​T	AGA​GCT​GCA​GAA​ATC​AGG​AAG​GCT
IL-11	AGA​TAT​CCT​GAC​ATT​GGC​CAG​GCA	ACT​TCA​GTG​ATC​CAC​TCG​CTT​CGT
IL-6	CCA​GCT​ATG​AAC​TCC​TTC​TC	GCT​TGT​TCC​TCA​CAT​CTC​TC
iNOS	GGT​CAG​AGT​CAC​CAT​CCT​CTT​TG	GCA​GCT​CAG​CCT​GTA​CTT​ATC
GAPDH	TCG​ACA​GTC​AGC​CGC​ATC​TTC​TTT	ACC​AAA​TCC​GTT​GAC​TCC​GAC​CTT

***Mouse***		
TRAP	ACA​TGA​CCA​CAA​CCT​GCA​G	CCT​CAG​ATC​CAT​AGT​GAA​ACC​G
MMP-9	GAT​CCC​CAG​AGC​GTC​ATT​C	CCA​CCT​TGT​TCA​CCT​CAT​TTT​G
CSTK	TGA​CCA​CTG​CCT​TCC​AAT​AC	CTC​TGT​ACC​CTC​TGC​ATT​TAG​C
Rankl	CGT​GCA​GAA​GGA​ACT​GCA​ACA​CAT	TTG​ATG​GTG​AGG​TGT​GCA​AAT​GGC
GAPDH	TCA​ACA​GCA​ACT​CCC​ACT​CTT​CCA	ACC​CTG​TTG​CTG​TAG​CCG​TAT​TCA

### Luciferase Assay

SaOS-2 cells were transfected using Genefectine transfection reagent (Genetrone Biotech, Seoul, Korea) in 24-well plates as per the manufacturer’s instructions. The 50 ng construct of Axin-2 or BRE (Addgene, Cambridge, MA, United States) and the same amount of Renilla luciferase thymidine kinase construct (Invitrogen) were used to estimate the luciferase activity.

Raw cells were transfected with nuclear factor of activated T-cells (NFATc) 1 construct (Addgene) by utilizing lipofectamine reagent (Invitrogen) as per the manufacturer’s protocol. Luciferase activities were evaluated by utilizing a dual-luciferase assay kit (Promega, Madison, WI, United States) as per the manufacturer’s instructions. Reporter activity was measured by a luminometer (GloMax, Promega, Sunnyvale, CA, United States). Each sample was normalized with Renilla luciferase activity.

### Protein Isolation and Western Blotting

Proteins from the cells were harvested in protease and phosphatase inhibitor cocktail containing RIPA buffer (20 mM Tris-HCl, pH 7.5, 200 mM NaCl, 1% Triton X-100, and 1 mM dithiothreitol) (Roche Diagnostics). After estimating the protein concentration in each sample by a protein assay kit (Bio-Rad Laboratories, Hercules, CA, United States), the total collected protein was loaded into SDS–polyacrylamide gel for electrophoresis. The membrane was probed with antibodies against COX-2 (Cell Signaling Technology, Danvers, MA) and β-catenin (Santa Cruz Biotechnology, Dallas, TX, United States). The β-actin antibody (Santa Cruz Biotechnology) was considered a loading control.

### Alkaline Phosphatase Activity (ALP) Assay

ALP activity of the osteoblasts was assayed, as described previously (Blum et al., 2001). In short, SaOS-2 cells were grown at a confluence of 9 × 10^4^ cells/well in 48-well plates. After 24 h, the cells were treated with the Ti CM. After treatment for 48 h, ALP activity was analyzed using the intensity of the luminescence reader GloMax as per the lab manual (Nam et al., 2017a).

### Alizarin Red S and Sirius Red Staining

In an osteogenic medium (DMEM with 10 mM β-glycerophosphate and 50 mg/mL ascorbic acid), SaOS-2 cells were stimulated to differentiate in a 24-well plate for 14 days. Every second day, the fresh control CM or Ti CM was replaced. The cells were fixed in 4% of paraformaldehyde solution for 1 h as per our lab protocol, and at room temperature, they were stained with Alizarin Red S solution (40 mM) (Sigma Aldrich) for 30 min. The microscope was used to picture the stained cells. For quantification of mineralization, initially, the stained cells were dissolved in 10% cetylpyridinium chloride (Sigma Aldrich) for 1 h, and after that, they were transferred to a 96-well plate for reading absorbance at 570 nm with a spectrophotometer (SoftMaxPro 5). For collagen staining, cell fixation was achieved by Bouin’s fluid (Sigma Aldrich) for 2 h. After that, the cells’ staining was performed with Sirius Red dye (1 mg/mL) (Sigma Aldrich), dissolved in saturated aqueous picric acid, for a minimum of 1 h. The stained cells were dissolved in 0.1 N sodium hydroxide by using a shaker for 30 min to measure the dye. Using a spectrophotometer at 550 nm, the optical density of the dissolved Sirius Red dye was estimated. 0.1 N sodium hydroxide was considered a blank.

### Detection and Quantification of TRAP-Positive Multinucleate Cells

RAW 264.7 cells were grown in a 24-well culture dish. Every second day, the medium was replaced. After 9 days of culture, the cell monolayer was gently washed twice with 1 mL PBS and fixed in 4% formaldehyde solution for 15 min. Then, the cells were washed with distilled water to remove fixation solution and stained with TRAP by using the Acid Phosphatase, Leukocyte kit (Sigma Aldrich) as per the manufacturer’s recommendations. Briefly, the cells were soaked in naphthol AS-BI 0.12 mg/mL, in the presence of 6.76 mm tartrate and 0.14 mg/mL fast garnet GBC, at 37 °C for 1 h in the dark. Then, the cells were washed and stained in hematoxylin for 30 s. Multinucleate cells positive for TRAP (MNCs, containing two or more than three nuclei) were counted using optical microscopy.

### TRAP Activity

TRAP activity was measured by the para-nitrophenylphosphate (pNPP) hydrolysis assay. After washing twice with PBS, the cells were solubilized with 200 µL solution of 0.1% (V/V) Triton X-100, following incubation for 1 h with 12.5 mm pNPP in 0.04 m tartaric acid and 0.09 m citrate (pH 5.8) at 37°C. By adding 5 mM NaOH, the reaction was stopped. At 405 nm, the absorbance was measured on an ELISA plate reader (Synergy HT, BioTek Instruments, Winooski, VT). The results were normalized with a total protein concentration of cultures.

### Antibody Array

The collected CM from FLSs or Ti particle–stimulated FLSs for 48 h was provided to the Ebiogen Company (Kyung Hee business center, Kyung Hee University, Seoul, Korea) for antibody array preparation and analysis. The explorer antibody microarray slide (Full Moon BioSystems, Sunnyvale, CA, United States) with 312 antibodies was treated with a blocking solution (30 mL) and kept on the shaker for 45 min at 37°C. After blocking, the slide was rinsed with 45 mL of double distilled water about 10 times. The labeled sample was dissolved in 5.3 mL of the coupling solution. The blocked array slide was incubated into a coupling dish containing a coupling mixture for 2 h at room temperature. After coupling, the array slide was bathed two times with 30 mL of 1x washing solution. Next, 30 μL of 0.5 mg/mL Cy3–streptavidin (GE Healthcare, Chalfont St. Giles, United Kingdom) was added in 30 mL of detection buffer and mixed by manual shaking. The array of slide scanning was carried out using the Revolution TM 4200 microarray scanner (Vidar Systems, Herndon, VA, United States) and ArraySifter Express 1.3 (Vidar Systems). The array slide was analyzed at 10 μm/pixel resolution and optimal laser power and in a photomultiplier tube. After getting the scanned image, it was gridded and evaluated with ArraySifter Express 1.3. The numeric data were assessed by utilizing Genowiz 4.0TM (Ocimum Biosolutions, India).

### ELISA

FLS cells were incubated with Ti particles (1:400) for 24 and 48 h, and the Ti CM was collected to identify sclerostin (SOST) concentration. The human SOST ELISA kit (R&D Systems, Minneapolis, MN, United States) was utilized for a quantitative measurement as per the manufacturer’s protocols.

### Neutralization of SOST in Ti CM

In order to neutralize SOST in the Ti CM, the Ti CM was pre-incubated with the anti-SOST (10 mg/mL) neutralization antibody (R&D Systems) at 37°C for 2 h and treated SaOS-2 cells for 48 h before analyzing ALP activity and Axin-2 or BRE reporter activity. For this experiment, non-immune isotype rat IgG was pre-incubated to the Ti CM (10 mg/mL) and was used as a control.

### Statistical Analysis

All the statistical data were assessed by GraphPad Prism 5.0 (GraphPad Software Inc., San Diego, CA, United States) and calculated by a two-tailed Student’s *t*-test. The values of *p* < 0.05, *p* < 0.01, and *p* < 0.001 were considered to indicate statistical significance.

## Results

### Ti Particles Stimulated Human FLS Produce Inflammatory and Immune Mediators

**FIGURE 1:**
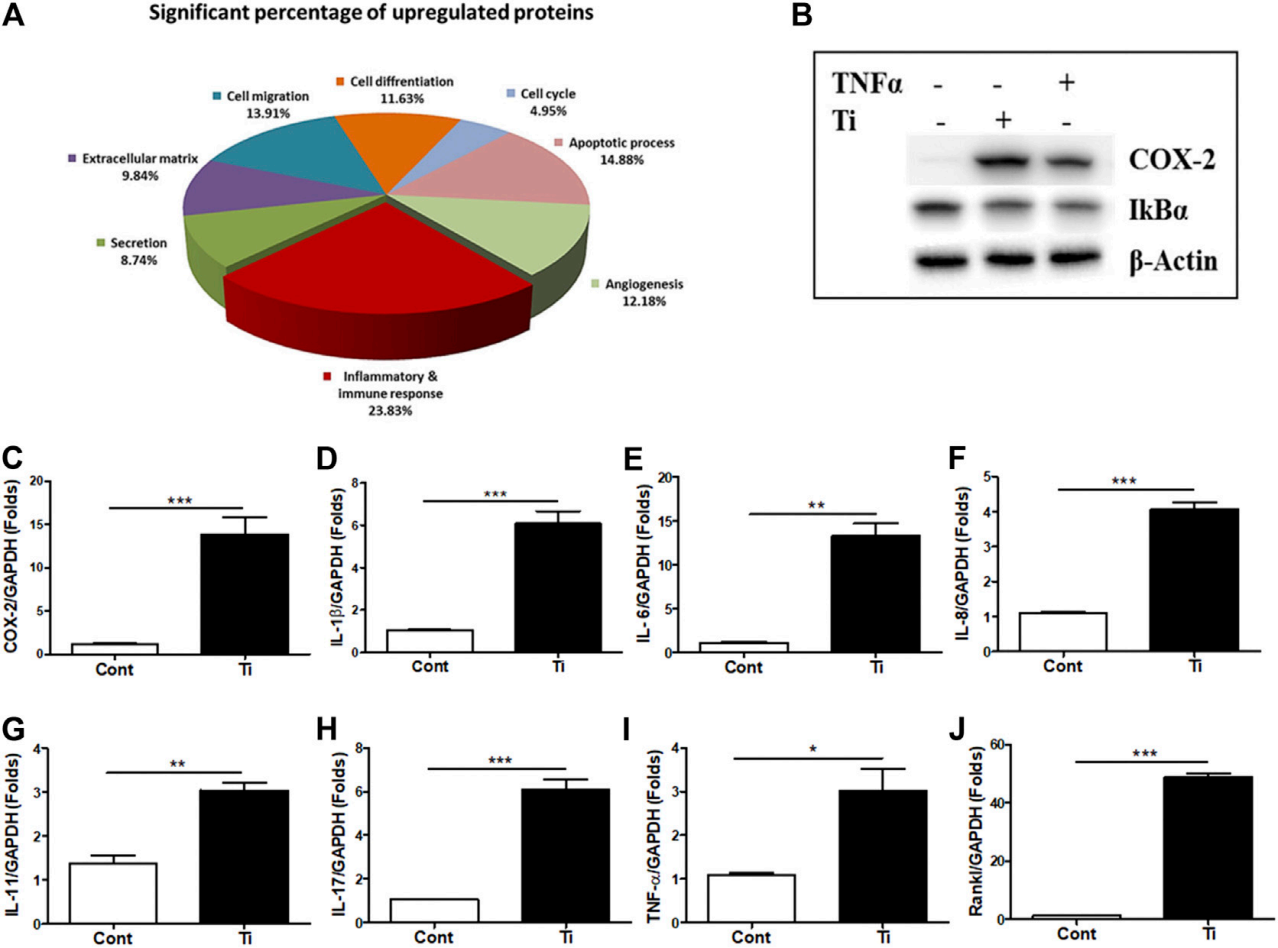
Increased expression of inflammatory and immune mediators in Ti particle–stimulated human FLSs. **(A)** Secretory protein array analysis of the Ti CM. Out of total secretory proteins, significantly upregulated secretory proteins are categorized and depicted as a percentage of their ratio to the control CM. **(B)** At 12 h of Ti particle treatment, FLSs showed induced expression of COX-2 and suppressed protein expression of IκBα. TNF-α is taken as a positive control. **(C–J)** Treatment of FLSs with Ti particles induced the mRNA expression of the inflammatory mediator, COX-2; pro-inflammatory cytokines, IL-1β, IL-6, IL-8, IL-11, IL-17, and TNF-α; and osteoclastogenic factor, Rankl. Data are shown as mean ± SD. Similar results were obtained in three independent experiments. *p < 0.05, **p < 0.01, and ***p < 0.001 vs. control.

Initially, to analyze any effect on cell viability and cytotoxicity, various cells’ ratios to Ti particles (1:100, 1:200, 1:300, and 1:400) were treated to FLS, and MTT and LDH assays were performed. Up to a ratio of 1:400, MTT and LDH assays demonstrated no significant effect of Ti particles on cell viability and cytotoxicity of human FLSs (Supplementary Figures S2A,B). To determine how FLSs might respond to Ti particle treatment, soluble proteins released in the culture medium were analyzed by the 312-antibody–coated antibody array chip. The amounts of secretory protein level measurements were clustered as a percentage of total regulated proteins: 10.79% extracellular matrix, 11.80% cell migration, 11.23% cell differentiation, 5.18% cell cycle, 11.45% apoptotic process, 11.30% angiogenesis, 32.83% inflammatory and immune response, and 14.42% secretion by considering the signal intensity of soluble proteins released in the Ti CM to the control CM (Supplementary Figure S2C). Furthermore, out of these total regulated proteins, the amount of significantly upregulated secretory proteins was further clustered as follows: 9.84% extracellular matrix, 13.91% cell migration, 11.63% cell differentiation, 4.95% cell cycle, 14.88% apoptotic process, 12.18% angiogenesis, 23.83% inflammatory and immune response, and 8.74% secretion (Figure 1A). It appeared that cumulatively, the inflammatory and immune response constituted about 25% of the proteins secreted by FLSs in response to Ti particles, indicating induction of inflammation. Expectedly, in a time-dependent manner, the treatment of FLSs with Ti particles increased the expression level of COX-2 (Supplementary Figure S2D). Also, the treatment of FLSs with Ti particles for 24 h suppressed the stability of IκBα, implicating activation of the NFκB signaling pathway (Figure 1B). Activation of the NFκB signaling pathway in FLSs has been found associated with the induction and expression of various inflammatory and immune response mediators (Makarov, 2001). On analyzing the mRNA expression by real-time PCR from Ti particle–stimulated FLSs for 24 h, the expression of COX-2 and pro-inflammatory cytokines such as IL-1β, TNF-α, IL-6, IL-8, IL-11, and IL-17 was increased (Figures 1C–I). Interestingly, a significant induction in the mRNA expression level of Rankl (∼50 folds) was detected in Ti-stimulated FLSs (Figure 1J).

### Ti CM Induced Osteoclastogenesis

**FIGURE 2:**
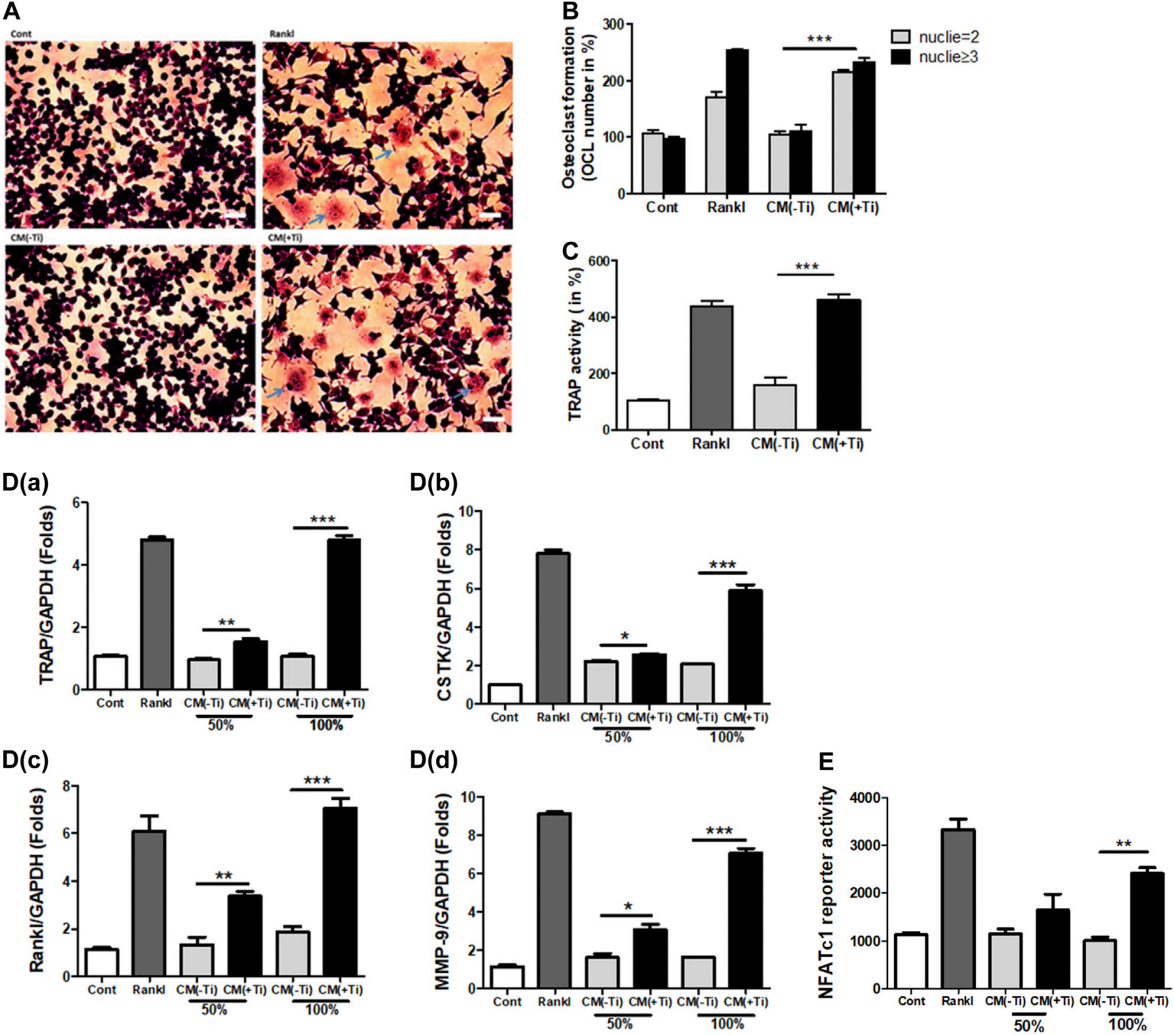
Ti CM induced osteoclastogenic differentiation in Raw 264.7 cells. **(A)** TRAP staining of osteoclasts (100×), osteoclast formation, and morphology were observed by light microscopy. **(B)** The number of TRAP-positive cells with two or more nuclei was counted after 9 days of Ti CM or Rankl (50 ng/mL) treatment of Raw 264.7 cells. Rankl treatment is taken as a positive control. **(C)** Quantification of TRAP activity. **D(a–d)** Ti CM induced the relative mRNA expression of osteoclast markers (TRAP, Rankl, MMP-9, and CSTK) after 9 days of Ti CM or Rankl treatment of Raw 264.7 cells. **(E)** NFATc1 reporter plasmid was transiently transfected to Raw 264.7 cells. The Ti CM treatment was done for 24 h, and reporter activity was analyzed from the cell lysate. Rankl treatment is taken as a positive control. Data are expressed as mean ± SD. *n* = 3. *p < 0.05, **p < 0.01, and ***p < 0.001 vs. control.

As an increase in the expression of cytokines such as IL-6 and Il-1β as well as osteoclastogenic factors such as Rankl was observed in the Ti particle–stimulated FLSs, a possibility of stimulating osteoclast formation was expected. IL-6 and IL-1β are known to involve in increasing the osteoclast formation, while Rankl is considered a primary factor responsible for inducing differentiation of osteoclast precursors to osteoclasts (Jandinski, 1988; Feng and Teitelbaum, 2013). Hence, at first, the mitogenic or cytotoxic effect of the Ti CM was examined on the murine macrophage cell line RAW 264.7. The Ti CM (25, 50, 75, and 100%) treated RAW 264.7 cells for 24, 48, and 72 h, and MTT and LDH assays were performed. Since a dose of 100% CM for 72 h of treatment showed no significant mitogenic and cytotoxic effects on RAW 264.7 cells, 100% CM was utilized for further experiments (Supplementary Figures S3A,B). To elucidate any effect of Ti CM on the formation of osteoclasts, RAW 264.7 cells were treated with the Ti CM alternatively for 9 days, and TRAP-positive cells were detected by TRAP staining as described in *Materials and Methods*. The Ti CM showed an increase in osteoclast formation by inducing multinuclear cell formations without affecting cell viability (Figures 2A–C). Moreover, the mRNA expressions of known osteoclast marker genes were examined by performing real-time PCR. Increased mRNA expression of TRAP, cathepsin K (CSTK), Rankl, and matrix metallopeptidase 9 (MMP-9) after treatment with the Ti CM for 9 days confirmed the effect of Ti CM in inducing osteoclastogenesis in RAW 264.7 cells [Figures 2D(a–d)]. Additionally, the Ti CM significantly induced the NFATc1 reporter activity in RAW 264.7 cells, which is sufficient to stimulate the transcriptional program, resulting in the attainment of the mature osteoclast cell phenotype (Figure 2E).

### Ti CM Suppressed the Osteogenic Parameters of Osteoblasts

**FIGURE 3:**
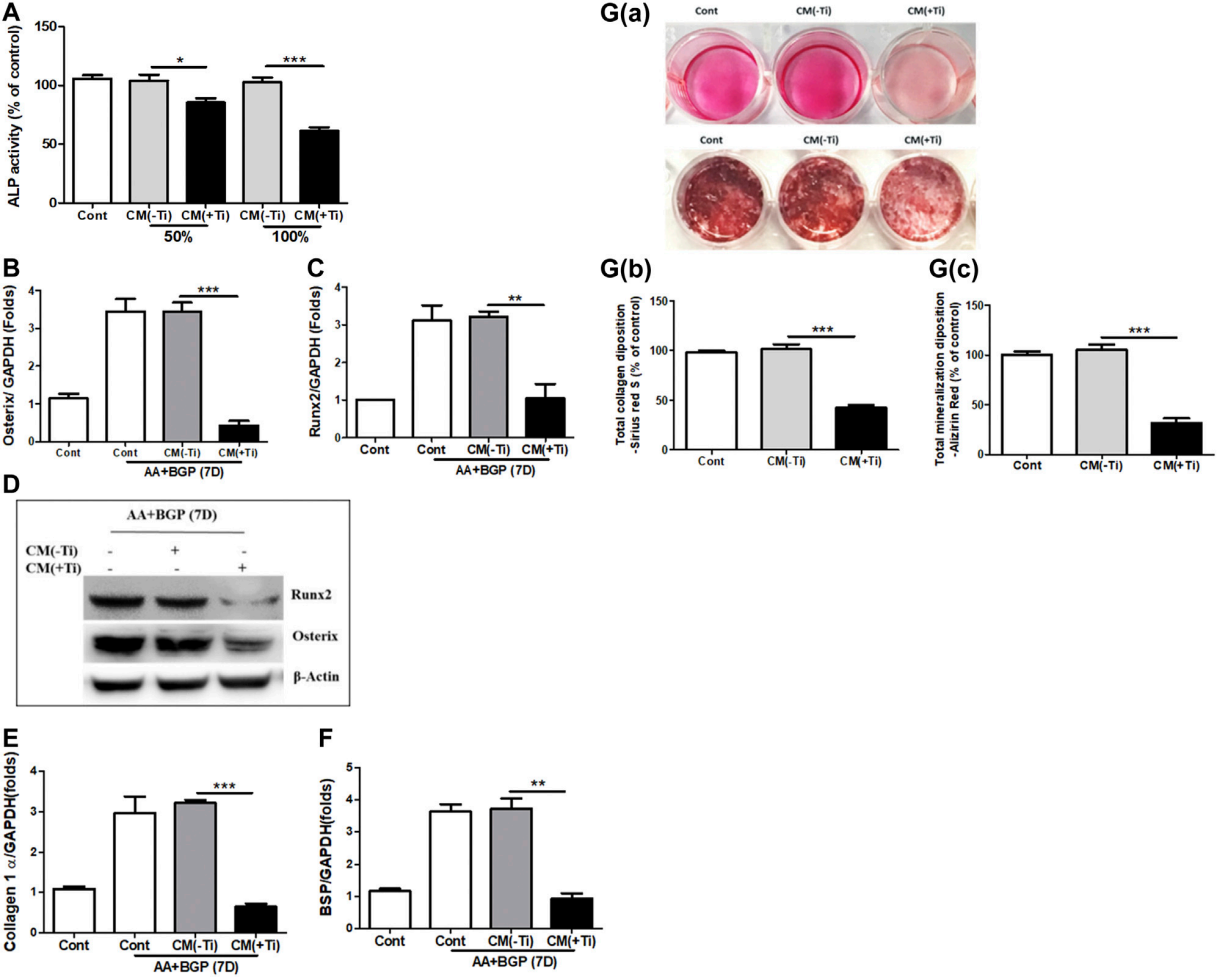
Ti CM suppressed osteogenic markers of osteoblasts. **(A)** ALP activity was measured with total lysate collected after 48 h of treatment with 50 or 100% Ti CM. SaOS-2 cells were grown under differentiation conditions as described in *Materials and Methods* for 7 days (D) and then treated with 100% Ti CM for 24 h. Transcripts of **(B)** Osterix, **(C)** Runx2, **(E)** collagen 1α, and **(F)** BSP were analyzed by real-time PCR with extracted total cellular RNA. The results shown are normalized to GAPDH levels. **(D)** Protein expressions of Runx2 and Osterix were analyzed by western blotting. β-Actin is used as a loading control. **(G(a))** Collagen synthesis and matrix mineralization were evaluated at day 14 by Sirius Red (upper lane) and Alizarin Red S (lower lane) staining (gross appearances), respectively, as described in *Materials and Methods*. **(G(b,c))** Quantitative analysis of Sirius Red S and Alizarin Red S staining. Data are shown as mean ± SD. Similar results were obtained in three independent experiments. *p < 0.05, **p < 0.01, and ***p < 0.001.

Next, any effect of Ti CM on the osteogenic activity of osteoblast cells was investigated. At first, MTT and LDH assays were performed to exclude the possibility of any mitogenic or cytotoxic effect of Ti CM on the human osteoblast cell line, SaOS-2. For this, different percentages of Ti CM (25, 50, 75, and 100%) treated SaOS-2 till 72 h. The results demonstrated no significant cytotoxic and mitogenic effects of Ti CM on SaOS-2 cells at any percentage of Ti CM treatment for 72 h (Supplementary Figures S3C,D). Thereafter, the effect of Ti CM on the early and late osteogenic parameters of osteoblasts was evaluated. ALP activity is considered an early differentiation osteogenic marker for osteoblasts. Thus, Ti CM treatment was done for 48 h, and ALP activity was analyzed. The results indicated a decline (more than 50%) in the ALP activity of SaOS-2 cells when treated with 100% Ti CM as compared to control (Figure 3A). As 100% Ti CM demonstrated no viability or cytotoxic effect on SaOS-2 cells and showed a maximum suppressive effect on ALP activity, all further experiments were carried out with 100% Ti CM. To further analyze the effect of Ti CM on the expression of other osteogenic markers, SaOS-2 cells were made to differentiate for 7 days by treating β-glycerophosphate (10 mM) and ascorbic acid (50 μg/mL) and, thereafter, were treated with the Ti CM for 24 h. mRNA was collected, and real-time PCR analysis was performed. The results demonstrated decreased mRNA expression of osteogenic transcriptional factors such as Osterix and Runx2 (Figures 3B,C). Additionally, the protein expression level of Runx2 and Osterix was also reduced by the Ti CM in osteoblasts (Figure 3D). The mRNA expression level of terminal osteogenic differentiation markers such as collagen 1 and BSP was also found to decrease (Figures 3E,F). Moreover, the effect of Ti CM on terminal differentiation markers for osteoblasts like collagen synthesis (Sirius Red S staining) and the process of mineralization (Alizarin Red S staining) were performed, as described in *Materials and Methods*. The results showed suppressed Sirius Red S and Alizarin Red S staining in the Ti CM–treated SaOS-2 cells [Figures 3G(a)]. Quantification of Sirius Red S and Alizarin Red S stains from the Ti CM–treated cultures showed a significant decrease in collagen synthesis and mineralization process for SaOS-2 cells [Figures 3G(b,c)].

### Ti CM Suppressed the WNT and BMP Signaling Pathways

**FIGURE 4:**
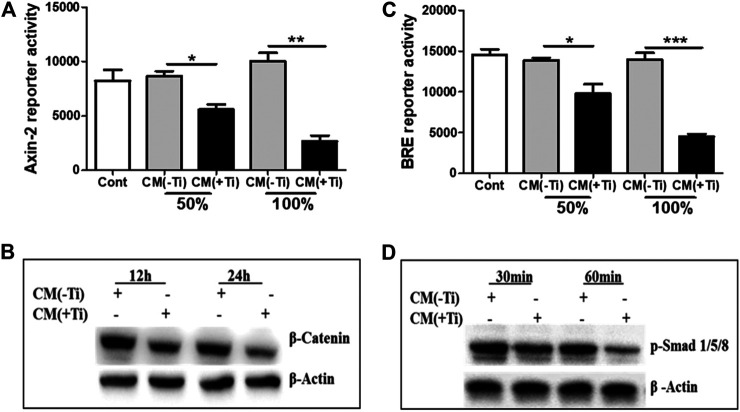
Ti CM suppressed WNT and BMP signaling pathways in osteoblasts. SaOS-2 cells were transiently transfected with the Axin-2 reporter construct for WNT signaling and BRE reporter constructs for BMP signaling and treated with different doses of Ti CM (50 and 100%). **(A)** Axin-2 reporter activity and **(B)** BRE reporter activity were performed with collected lysate. **(C)** Protein expression of β-catenin after 12 and 24 h of Ti CM treatment and **(D)** protein expression of *p*-Smad 1/5/8 after 30 and 60 min of Ti CM treatment were evaluated by western blotting. β-Actin is used as a loading control. Data are shown as mean ± SD. Similar results were obtained in three independent experiments. *p < 0.05, **p < 0.01, and ***p < 0.001 vs. control.

Since Ti CM treatment of osteoblast cells reduced osteogenic markers, it was assumed that the Ti CM might have an inhibitory effect on vital osteogenic signaling pathways such as WNT and BMP, required for osteoblast differentiation. Hence, to investigate whether the Ti CM can affect WNT and BMP signaling pathways, luciferase reporter assays were used. SaOS-2 cells were transfected with the reporter construct of Axin-2-luc or BRE-luc for WNT and BMP pathways, respectively. After 24 h of treatment with the Ti CM, a significant decline in the basal level of both Axin-2-luc and BRE-luc luciferase activity exemplifies the inhibitory role of Ti CM in WNT and BMP signaling pathways (Figures 4A,C). During the process of osteoblast differentiation, canonical WNT signaling gets activated over the stabilization of β-catenin, while phosphorylation of Smad 1/5/8 is accountable for activation of BMP signaling activity (Lerner and Ohlsson, 2015; Mulloy and Rider, 2015). Hence, to investigate precisely the inhibitory consequence of Ti CM on WNT and BMP signaling pathways, the protein expression of β-catenin and phosphorylation of Smad 1/5/8 were analyzed by performing western blotting. The obtained results indicated that Ti CM treatment suppressed the stability of β-catenin after 12 and 24 h compared to control (Figure 4B). In the same way, a decrease in the phosphorylation level of Smad 1/5/8 was noted after 30 and 60 min compared to control (Figure 4D).

### Exogenous Stimulation With Wnt3a and Bmp-2 Proteins Fails to Recover Suppressed Osteogenesis

**FIGURE 5:**
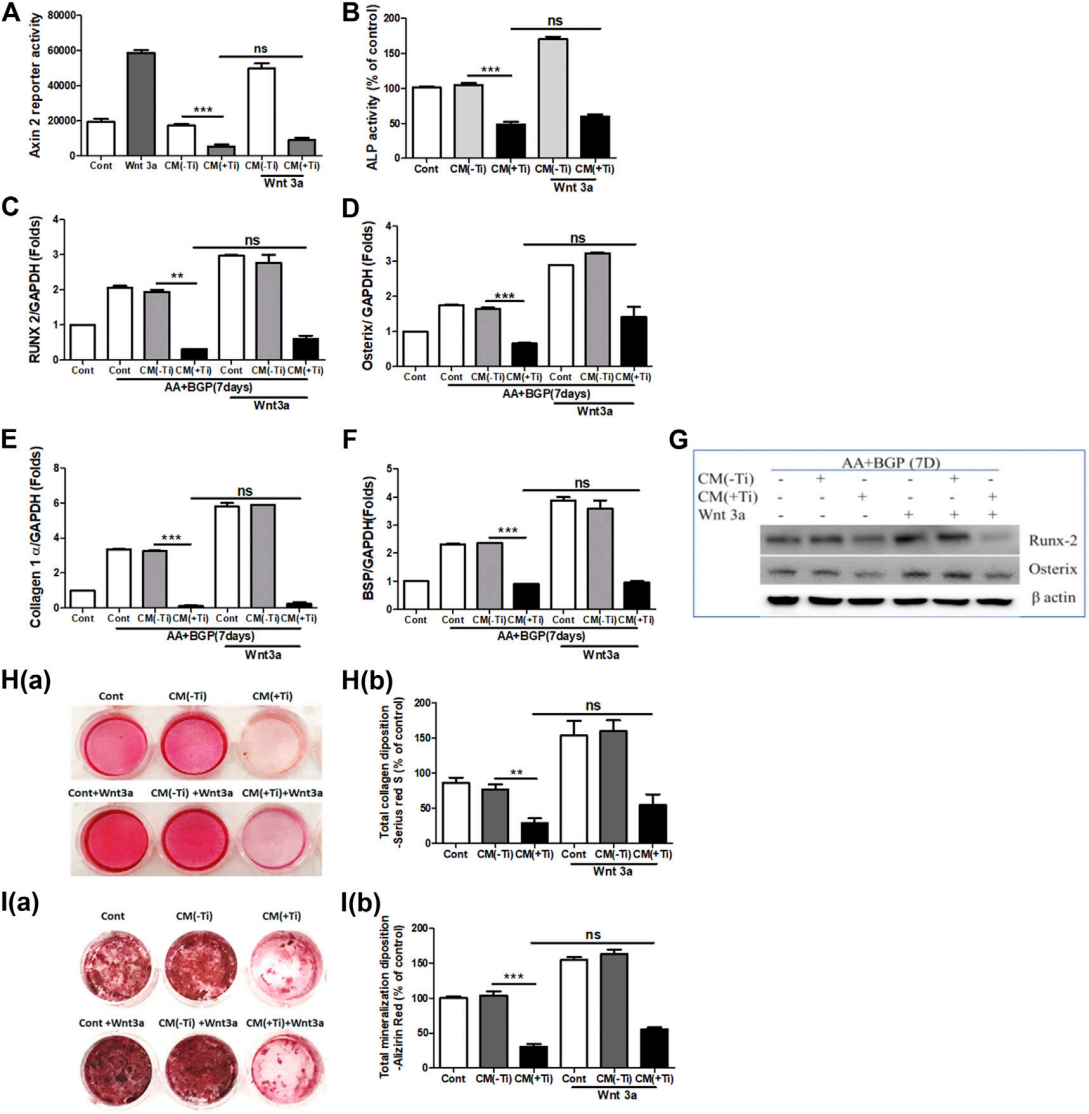
Ti CM inhibits osteogenic induction after stimulation of osteoblasts by exogenous Wnt3a. The SaOS-2 cell was pre-incubated with the Ti CM for 30 min and thereafter stimulated with recombinant Wnt3a along with the Ti CM. **(A)** Cell lysates from Ti CM–treated SaOS-2 cells (Axin-2 reporter construct transfected) for 24 h were collected, and reporter activity was analyzed. **(B)** ALP assay was performed after 48 h of treatment. **(C–F)** SaOS-2 cells were grown under differentiation conditions as described in *Materials and Methods* for 7 days **(D)** and then treated with 100% Ti CM for 24 h. Relative mRNA expressions of Osterix, Runx2, collagen 1α, and BSP were evaluated by real-time PCR. The results shown are normalized to GAPDH levels. **(G)** Protein expressions of Runx2 and Osterix were analyzed by SDS gel electrophoresis. β-Actin is used as a loading control. **(H)** Collagen synthesis and (**I**) mineralization were evaluated after 14 days of treatment, by Sirius Red and Alizarin Red S staining, respectively. Collagen deposition depicted as **(H(a))** gross appearances. **(H(b))** Quantitative analysis of Sirius Red S staining. The effect of Ti CM on mineralization is depicted as **(I(a))** gross appearances. **(I(b))** Quantitative analysis of Alizarin Red S staining. Data are shown as mean ± SD. Similar results were obtained in three independent experiments. *p < 0.05, **p < 0.01, ***p < 0.001, and ns (no significance) vs. control.

**FIGURE 6:**
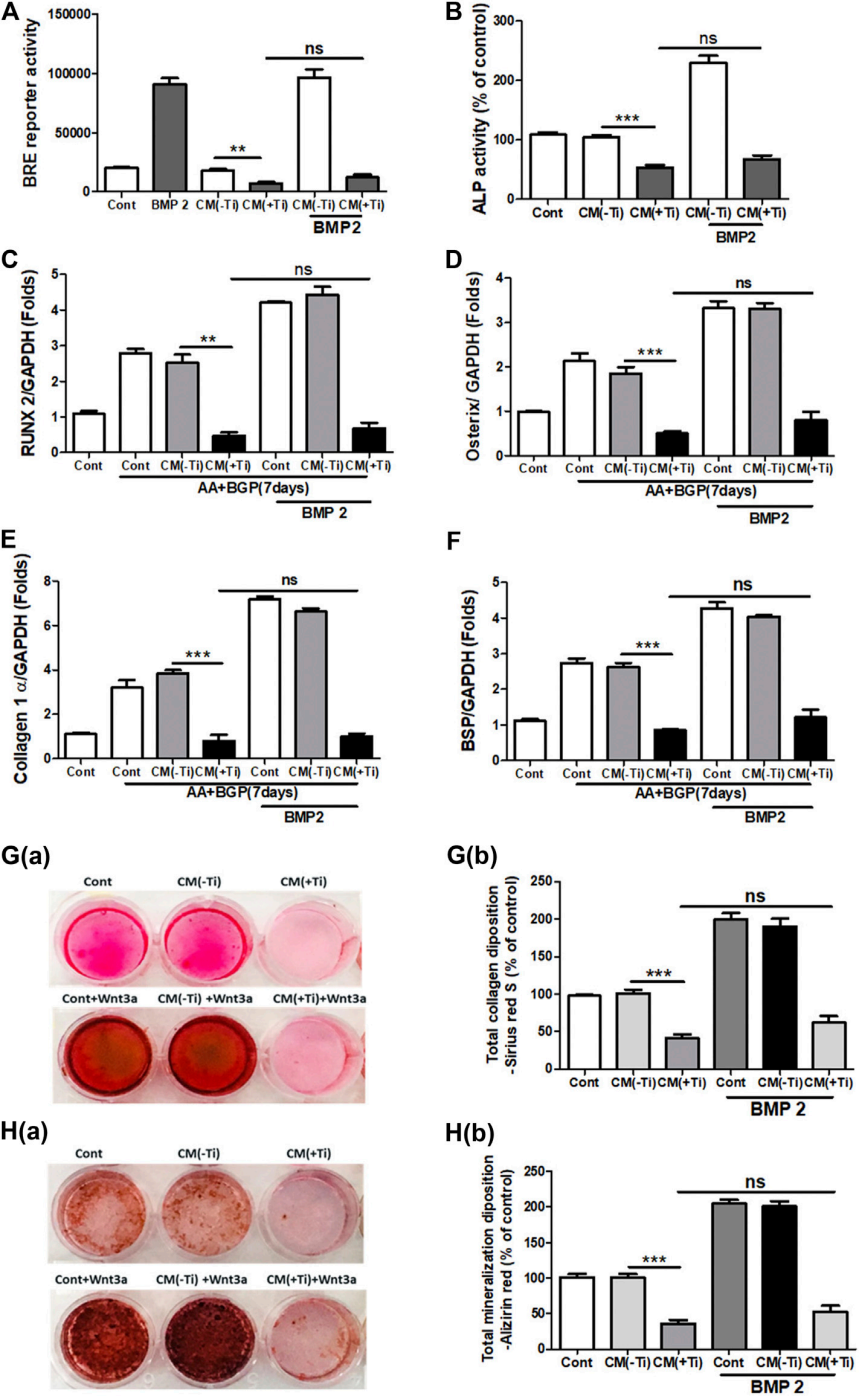
Ti CM inhibits osteogenic induction after stimulation of osteoblasts by exogenous Bmp-2. The SaOS-2 cell was pre-incubated with the Ti CM for 30 min and thereafter stimulated with recombinant Bmp-2 along with the Ti CM. **(A)** Cell lysates from Ti CM–treated SaOS-2 cells (Axin-2 reporter construct transfected) for 24 h were collected, and reporter activity was analyzed. **(B)** ALP assay was performed after 48 h of treatment. **(C–F)** SaOS-2 cells were grown under differentiation conditions as described in *Materials and Methods* for 7 days **(D)** and then treated with 100% Ti CM along with Bmp-2 for 24 h. Relative mRNA expressions of Osterix, Runx2, collagen 1α, and BSP were evaluated by real-time PCR. The results shown are normalized to GAPDH levels. **(G)** Collagen synthesis and **(H)** mineralization were evaluated after 14 days of treatment by Sirius Red and Alizarin Red S staining, respectively. **(G(a))** Collagen deposition gross appearances. **(G(b))** Quantitative analysis of Sirius Red S staining. The effect of Ti CM on mineralization is depicted as **(H(a))** gross appearances. **(H(b))** Quantitative analysis of Alizarin Red S staining. Data are shown as mean ± SD. Similar results were obtained in three independent experiments. *p < 0.05, **p < 0.01, ***, *p* < 0.001, and ns (no significance) vs. control.

To confirm the involvement of WNT and BMP signaling pathways in Ti CM–induced impaired osteogenesis, we further attempted to recuperate the Ti CM–suppressed WNT and BMP signaling pathways in SaOS-2 cells by stimulating with recombinant Wnt3a (25 ng/mL) and Bmp-2 (50 ng/mL), exogenously. The expectation was to restore the suppressed osteogenic (WNT and BMP) signaling and to reinstate the impeded osteogenic activity in Ti CM–treated osteoblasts. For this, SaOS-2 cells, transiently transfected with either Axin-2 or BRE luciferase plasmids, were co-treated with the Ti CM and Wnt3a or Bmp-2 for 24 h, and luciferase reporter activity was analyzed. Exogenous stimulation failed to induce Axin-2 or BRE luciferase activity in Ti CM–treated cells, whereas increased luciferase activity was detected in control CM–treated cells as anticipated (Figures 5A and 6A). In order to verify the inhibitory role of Ti CM in osteogenic signaling pathways, the Ti CM along with Wnt3a or Bmp-2 co-treated SaOS-2 cells, and ALP activity was analyzed. Exogenous stimulation by Wnt3a and Bmp-2 failed to demonstrate any recovery of ALP activity in the presence of the Ti CM (Figures 5B and 6B). Furthermore, SaOS-2 cells were induced to differentiate as described earlier, and any stimulatory effect of Wnt3a and Bmp-2 proteins, in the presence of the Ti CM, on the mRNA expression level of osteogenic markers such as Runx2, Osterix, collagen 1α, and BSP was analyzed. The results showed no induction in the mRNA expression levels of these osteogenic markers in the presence of the Ti CM (Figures 5C–F, 6C–F). Moreover, no induction in Runx2 and Osterix protein levels was observed in the case of Wnt3a co-treatment with the Ti CM (Figure 5G). Lastly, the exogenous stimulatory effect of Wnt3a and Bmp-2 was analyzed on late osteogenic markers such as collagen synthesis and mineralization. Co-treatment with Wnt3a along with the CM induced collagen synthesis (Sirius Red S staining) and mineralization (Alizarin Red S) in SaOS-2 cells in the control CM but failed in the case of the Ti CM [Figures 5H(a,b) and I(a,b)]. Similar inhibition of collagen synthesis and mineralization was observed for the co-treatment with Bmp-2 along with the Ti CM [Figures 6G(a,b) and H(a,b)].

### SOST Accounts for the Impeded Osteogenesis

**FIGURE 7:**
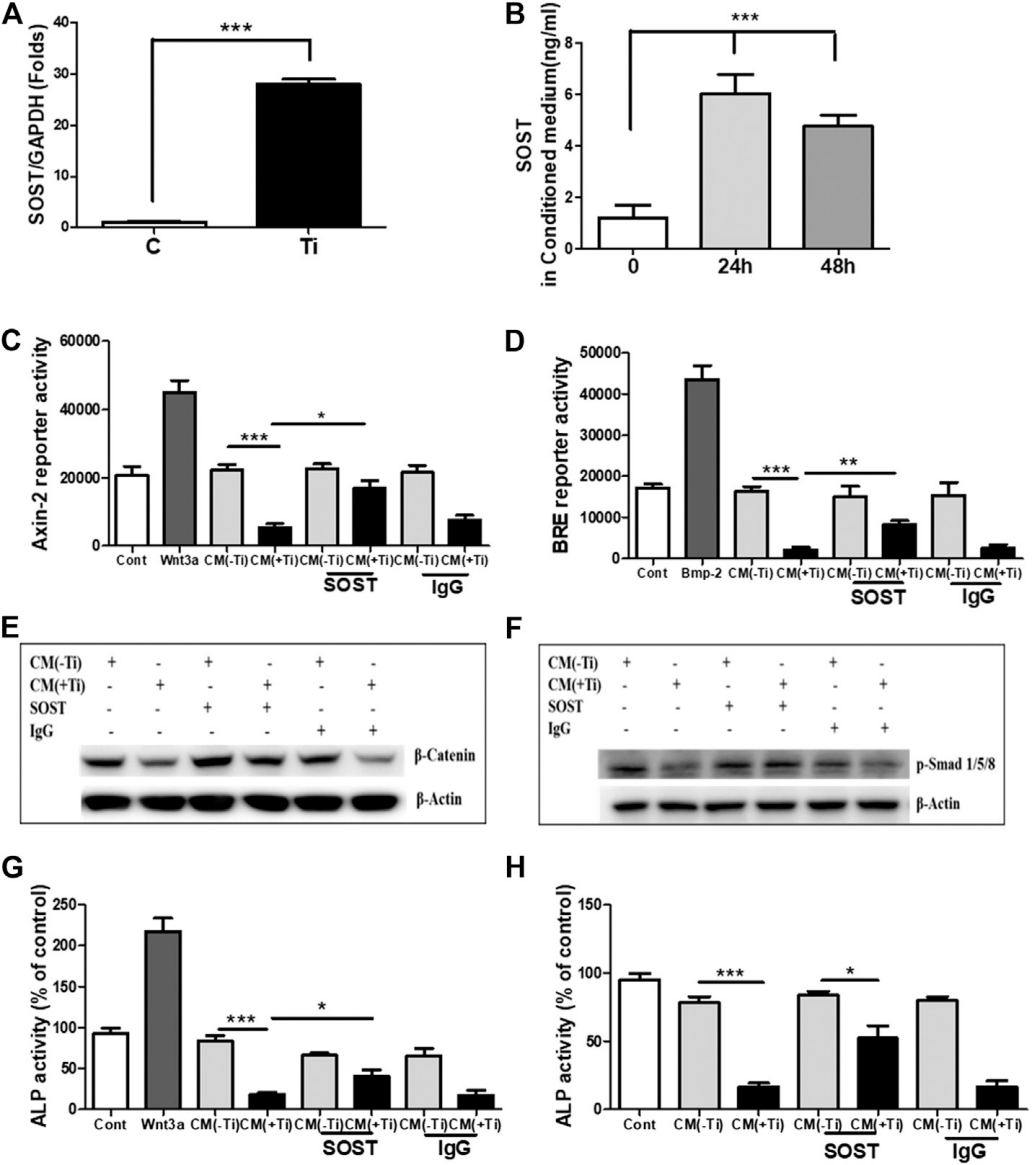
SOST in the Ti CM is responsible for suppressed WNT and BMP signaling and ALP activity in osteoprogenitors. **(A)** Treatment of FLSs with Ti particles induced the mRNA expression of SOST (∼40 folds) in FLSs. **(B)** ELISA showed induced SOST release in the CM of FLSs after 24 h (∼6 ng/mL) and 48 h (∼5 ng/mL) of Ti particle treatment. **(C)** Antibodies against SOST were incubated in the Ti CM for 2 h prior to treatment of SaOS-2 cells. After 24 h of treatment, Axin-2 reporter activity and **(D)** BRE reporter activity were analyzed. **(E)** Protein expression of β-catenin was analyzed by SDS gel electrophoresis. **(F)** Stability of *p*-Smad 1/5/8 protein expression was analyzed by SDS gel electrophoresis. **(G)** Changes in ALP activity of SaOS-2 were analyzed after 48 h of treatment. **(H)** Antibodies against SOST were incubated in the Ti CM for 2 h prior to treatment of primary human osteoblast cells. After 48 h of treatment, changes in ALP activity were analyzed. Data are shown as mean ± SD. Similar results were obtained in three independent experiments. *p < 0.05, **p < 0.01, and ***p < 0.001 vs. control.

In lieu of the above results, the next intention was to identify the secreted molecules from Ti-stimulated FLSs, which might explain the inhibitory role of Ti CM in osteogenic signals and osteogenic activity of osteoblasts. Several mechanisms exist for the regulatory mechanism of WNT and BMP signaling pathways, and one of them is through secreted extracellular antagonists (Malinauskas and Jones, 2014; Lerner and Ohlsson, 2015; Mulloy and Rider, 2015). Several extracellular antagonists for the WNT [SOST, Dickkopf (DKK), secreted frizzled-related protein (sFRP), Wnt inhibitory factor (WIF), and Wise] and BMP (noggin, chordin, follistatin, and SOST) signaling pathways are known. On analyzing the mRNA expression of various antagonists to the WNT signaling pathway in Ti particle–stimulated FLSs for 24 h, increased expression of antagonists such as DKK and sFRP families and SOST was observed. Among various screened antagonists, inductions of DKK2, sFRP3, and sFRP5 were significantly higher (>20 folds) (Supplementary Figures S4A–C). Moreover, expression levels of the known antagonists of BMP signaling (noggin, chordin, and follistatin) were also found to be increased (Supplementary Figures S4D–F). Among these overexpressed antagonists, SOST is a negative regulator of both WNT (Collette et al., 2010) and BMP (Krause et al., 2010) signaling pathways, and thus, its extent of release in the Ti CM needs to be evaluated. For this, the mRNA expression level of SOST was measured by real-time PCR after 24 h of Ti particle stimulation, and the concentration of SOST in the Ti CM after 24 and 48 h of Ti particle stimulation was measured by ELISA. The mRNA expression of about 40 folds for SOST was observed after Ti particle stimulation in FLSs (Figure 7A). Moreover, ELISA results showed an increase in secreted SOST in the Ti CM (∼6 ng/mL) compared to control at 24 h (Figure 7B).

Significant release of SOST in the Ti CM and its ability to inhibit WNT and BMP signaling pathways in osteoblasts make it an ideal candidate for studying the extent of its involvement in inhibiting the osteogenic activity of osteoblasts. Thus, to account for the involvement of SOST, an anti-SOST antibody was used to neutralize SOST in the Ti CM, and the extent of recovery on WNT and BMP signaling pathways and ALP activity was observed in osteoblasts. The Ti CM was incubated with the SOST-neutralizing antibody for 30 min prior to treating SaOS-2 cells, and the effect was analyzed by luciferase reporter assays (for WNT and BMP signaling activity) and ALP activity. The results showed partial recovery of inhibited Axin-2 and BRE reporter activity after blocking of SOST in the Ti CM, clearly suggesting a recovery of WNT and BMP signaling pathways after neutralization of SOST in the Ti CM (Figures 7C,D). A confirmation for the recovery of WNT and BMP signaling activity after neutralization of SOST was further affirmed by the stabilization of β-catenin and phosphorylation of Smad 1/5/8 molecules, respectively, by western blotting (Figures 7E,F). The neutralization of SOST in the Ti CM resulted in the recovery of stability of the β-catenin protein as well as in the recovery of the phosphorylation of Smad 1/5/8 molecules. Moreover, the neutralization of SOST in the Ti CM also resulted in partial restoration of the ALP activity of SaOS-2 cells (Figure 7G). In order to validate whether the effect of Ti CM is reproducible in other *in vitro* osteoblast models, cultures of primary human osteoblasts were established. Treatment with the Ti CM suppressed the ALP activity (around 70%) even in primary human osteoblasts, similar to SaOS-2 cells. Moreover, treatment with the Ti CM having SOST neutralized by the anti-SOST antibody showed a partial recovery of ALP activity in primary human osteoblasts, validating our results from SaOS-2 cells (Figure 7H).

## Discussion

After TJA, regular usage of implants is often associated with their failures in clinical settings. A release of the substantial quantity of wear debris, forming the mechanical abrasion of implant surfaces, usually elicits an inflammatory response in a diverse variety of cells present at the bone–implant bed in synoviums such as FLSs, macrophages, osteoblasts, osteoclasts, and dendritic cells (Mbalaviele et al., 2017). Our previous studies have highlighted the importance of cellular interaction among various cell types in affecting the low bone-forming ability of osteoblasts during periprosthetic-like conditions. It was observed that the secretion of TNF-α from Ti particle–stimulated macrophages inhibits the osteogenic ability of osteoprogenitors by inducing autocrine secretion of the WNT and BMP signaling pathways’ antagonist, SOST, from osteoblasts (Lee et al., 2012). Among various cell types present at the periprosthetic membrane, FLSs constitute the majority and have been shown to get affected by wear debris from the articulating prosthetic surfaces. Hence, the present study was aimed to observe any role of Ti particle–stimulated FLSs in enhanced osteoclastogenesis or impeded osteogenesis as often observed during wear debris–induced osteolysis. For this, FLSs isolated from human synovial tissue samples were characterized (Supplementary Figure S1) and utilized to study the effect of wear debris (Ti particles). Wear debris has been known to elicit an inflammatory response in FLSs by causing an efflux of pro-inflammatory mediators such as IL-6, TNF-α, IL-1β, COX-2, and prostaglandin E2 (PGE2) (Bukata et al., 2004; Fujii et al., 2011). In accordance with these studies, stimulation of human FLSs by Ti particles induced the expression of COX-2 and activated the NFκB signaling pathway (Figure 1). Moreover, increased mRNA expression of inflammatory and osteoclastogenic mediators such as IL-1β, TNF-α, IL-6, IL-8, IL-11, IL-17, and Rankl was observed in Ti particle–stimulated FLSs (Figure 1).

Wear debris–stimulated FLSs have been known to secrete bone-resorbing factors and support osteoclastogenesis (Koreny et al., 2006). Since Ti particle–stimulated FLSs demonstrated increased expression of pro-inflammatory mediators, the effect of these secretions needed to be defined. The Ti CM increased the mRNA expression of osteoclastic factors such as TRAP, Rankl, CSTK, and MMP-9 in the macrophage cell line, RAW 264.7 cells. Moreover, stimulation by Ti CM increased the osteoclastogenic marker, TRAP activity (TRAP staining), and induced the formation of multinucleated mature osteoclasts in RAW 264.7 cells (Figure 2). These results validated the presence of secreted osteoclastogenic mediators from Ti-stimulated FLSs in the Ti CM and confirmed the involvement of FLSs in increased osteoclastogenic activity during wear debris–induced osteolysis.

During periprosthetic osteolysis, apart from enhanced osteoclastogenesis, impeded osteogenic activity is another major reason for bone erosion after TJAs (Lee et al., 2012; Nam et al., 2017b). Lately, fibroblasts derived from the interfacial membranes of patients with failed implant replacements have been reported to secrete bone-resorbing MMPs and mediator(s), which may cause suppressed collagen synthesis in osteoblasts (Yao et al., 1995). However, there are no other reports that could provide an insight into the role of FLSs in the impeded osteogenic activity of osteoblasts. Hence, in order to observe any role of secreted molecules by FLSs in the Ti CM in the osteogenic ability of osteoblasts, osteogenic parameters of SaOS-2 cells were observed after treatment with the Ti CM. It was interesting to note that secreted factors present in the Ti CM suppressed early (ALP activity, transcription factors: Runx2 and Osterix) and late (BSP, mineralization, and collagen synthesis) differentiation markers for osteoblasts (Figure 3). During skeletal development, cell proliferation, differentiation, and survival of the osteogenic lineage are under the tight regulatory control of WNT and BMP signaling pathways (Sharma et al., 2013; Mulloy and Rider, 2015; Karner and Long, 2017). As the Ti CM suppressed the osteogenic activity of osteoblasts, it was assumed that secretory factors present in the Ti CM might have an inhibitory role in the bone signaling pathways such as WNT and BMP. Treatment with the Ti CM suppressed basal WNT and BMP signaling activity in osteoblasts, implicating an inhibitory role of secreted factors in the CM in osteogenic signaling pathways (Figure 4). Moreover, exogenous treatment by recombinant Wnt3a and Bmp-2 proteins failed to rescue the suppressive effect of Ti CM on both WNT and BMP signaling pathways and osteogenic markers, validating the inhibitory role of secretory factors in the CM for their ability to inhibit WNT and BMP signaling pathways and osteogenic activity of osteoblasts (Figures 5 and 6).

One of the regulatory mechanisms through which both WNT and BMP signaling pathways are managed is via secreted antagonists (Sharma et al., 2015; Karner and Long, 2017). Under inflammatory conditions like rheumatoid arthritis (RA), FLSs have been shown to possess the ability to secrete WNT antagonists such as DKK1 and SOST (Yeremenko et al., 2015; Wehmeyer et al., 2016). Since Ti particles also induced inflammatory response in FLSs, it raises the possibility of the secretion of WNT and BMP signaling antagonists in the Ti CM. Screening for any upregulated expression of WNT and BMP signaling antagonists after treatment of FLSs with Ti particles revealed that the mRNA expression of WNT antagonists, DKK2, sFRP3, sFRP5, and SOST, was increased. For the BMP signaling pathway, the mRNA expression of antagonists such as noggin, follistatin, SOST, and chordin was found to be elevated (Supplementary Figure S4 and Figure 7). Among all these antagonists, only SOST is known to inhibit both WNT and BMP signaling pathways (Krause et al., 2010; Lee et al., 2012). SOST can bind to the lipoprotein receptor–related protein 5 or 6 co-receptor (LRP5/6), preventing the WNT ligand from binding to the frizzled-LRP complex receptor for activating the WNT signaling pathway (Li et al., 2005). In contrast, in the case of BMP signaling, SOST has the ability to bind to BMP molecules and lead to sequestration and intracellular proteasomal degradation, inhibiting the BMP signaling pathway. To validate the involvement of SOST, SOST was neutralized by SOST-specific antibodies, and some effect on the recovery of osteogenesis was observed. The results demonstrated partial recovery of both WNT and BMP signaling pathways after neutralization of SOST in the Ti CM. Moreover, the neutralization of SOST in the Ti CM caused partial restoration of the ALP activity of osteoblasts. These results confirm and validate the inhibitory role of SOST, secreted by Ti particle–stimulated FLSs, in the Ti CM, impeding the osteogenic activity of osteoblasts by abrogating WNT and BMP signaling pathways in osteoblasts (Figure 7).

However, failure to obtain a complete recovery of WNT and BMP signaling pathways as well as ALP activity after neutralization of SOST in the Ti CM raises the possibility of the existence of other factors or mechanisms which require further verification. An increase in the expression of other WNT and BMP antagonists was observed after stimulation of FLSs by Ti particles (Figure 7). Since SOST had the ability to inhibit both WNT and BMP signaling pathways, its role in suppressed osteogenic activity was studied. However, it is possible that other antagonists of WNT and BMP signaling pathways might be able to contribute toward the inhibition of both the osteogenic signaling pathways and, finally, the osteogenic activity of osteoblasts. Thus, further studies would be required to delineate the roles of these antagonists, which might explain the partial recovery of osteogenic activity after neutralization of SOST only. Another possibility that might contribute to the complete recovery of suppressed osteogenic activity by the Ti CM from FLSs is that, under inflammatory conditions, enhanced secretion of cytokine-like TNF-α might be responsible for inducing secretion of SOST from osteoblasts, which in turn can act in an autocrine manner to affect its own osteogenic activity (Lee et al., 2012). Moreover, TNF-α itself has been shown to possess either a stimulatory or an inhibitory effect on bone formation and thus might be another factor that needs to be investigated (Gerstenfeld et al., 2001). Thus, combination therapy of inhibition of inflammation to control the secretion of cytokines like TNF-α along with the suppression of SOST can be an effective therapeutic option in preventing or delaying periprosthetic osteolysis. Nevertheless, the role of various cell types in the secretion of inflammatory factors like TNF-α and the molecules like SOST that can directly affect the bone-forming ability of osteoblasts needs more detailed studies.

A clear insight into the molecular interactions among the various cell types in the synovium around the prosthesis is very much required, and our understanding of this phenomenon is currently very limited. Though the study was able to identify the secretion of anti-osteogenic factors from Ti-stimulated FLS *in vitro* models, further studies based on more relevant *in vivo* models would be required to affirm the findings. However, the release of SOST by osteoblasts as well as FLSs during wear debris–induced inflammation highlights the importance of SOST in the synovial space, and in what magnitude it may affect the bone formation event during periprosthetic osteolysis requires further investigation. Though we observed the release of SOST in Ti particle–stimulated FLSs, the exact mechanism responsible for the release of SOST was not studied. There is a lack of studies that could explain the mechanism of release of SOST in FLSs. However, in the bone, several studies have highlighted that factors such as cytokines, growth factors, and hormones can regulate the release of SOST at the transcription level by regulating the proximal promoter and the distal enhancer *ECR5* (Sebastian and Loots, 2017). Thus, a similar mechanism might be expected to regulate the release of SOST in FLSs; however, further studies are required to delineate this mechanism in FLSs. Wear debris has been known to stimulate the cells (osteoblasts/macrophages) by several mechanisms, which can be phagocytic and non-phagocytic (Zhang et al., 2020). Here, we have just studied the stimulatory effect of Ti particles on FLSs and their participatory role in suppressed osteolysis during periprosthetic osteolysis. Since internalization of wear particles is a prerequisite for a cellular reaction to particles (for example, inflammatory reaction and cytotoxicity), the mechanism of internalization can be considered a potential target for particle-induced osteolysis. Hence, further studies are required to elucidate the mechanism of uptake of Ti particles by FLSs. Moreover, the research addressing the effect of size and shape of Ti particles will further help us understand the overall mechanism of action of Ti particles on FLSs.

Recently, the human SOST monoclonal antibody (romosozumab) has been approved for the treatment of osteoporosis in postmenopausal women at high risk for fracture. This humanized monoclonal antibody is designed to work by inhibiting the activity of sclerostin (SOST), simultaneously resulting in increased bone formation and, to a lesser extent, decreased bone resorption (AMGEN, 2020). A similar approach to inhibit SOST would be beneficial in treating the impeded bone formation during periprosthetic osteolysis. However, for application in clinical settings, a more rigorous approach would be required to understand the cross-talk among the various cell types in the tissue implant interface so that the consequence of utilizing the drugs targeted toward the identified factors could be assessed. Furthermore, owing to the lubricative ability of synovium, a retrieval of it has been suggested during total hip replacements to reduce the friction between the various parts of the prosthesis (Park et al., 2014). However, under inflammatory conditions, considering the release of antagonists (like SOST) of bone-forming signaling from the various cell types such as osteoblasts and fibroblasts in the synovium, retrieval of synovium for the lubricating purpose is thus debatable. Therefore, the clinicians should be careful in considering the consequence of retrieving the synovium with respect to bone formation after replacement surgeries. Moreover, SOST-induced impaired osteogenesis is not specific only to Ti implant debris. Recently, wear debris–like lipopolysaccharide-doped polyethylene particles and β-tricalcium phosphate have also been shown to exert SOST-mediated impaired osteogenesis (Liu et al., 2012; Zhang et al., 2012). Thus, targeting SOST in the clinical settings to prevent impeded bone formation during periprosthetic osteolysis might be a rewarding option.

## Conclusion

Taken together, it may be concluded that wear debris–stimulated FLSs play a critical role in affecting the bone remodeling process during periprosthetic osteolysis by influencing two processes, osteoclastogenesis and osteoblastogenesis. In response to wear debris, FLSs are capable of releasing pro-inflammatory mediators that can affect and induce osteoclastogenesis as well as the secretion of certain factors like SOST, which can even affect the bone-forming ability of osteoblasts. This study provides an insight into the regulatory mechanism that might explain the bone defects as observed during inflammatory conditions such as RA and periprosthetic osteolysis and highlights the role of FLSs in secreting bone-inhibiting molecules like SOST, which might be the suitable therapeutic target for treating the impaired bone formation in wear debris–induced osteolysis.

## Data Availability

The raw data supporting the conclusions of this article will be made available by the authors, without undue reservation.
