# Supplementary material for: Sclerostin-Mediated Impaired Osteogenesis by Fibroblast-Like Synoviocytes in the Particle-Induced Osteolysis Model
Source: Front Mol Biosci. 2021 Jun 23;8:666295. doi: 10.3389/fmolb.2021.666295 (PMC8260695; doi:10.3389/fmolb.2021.666295)
Supplement: Supplementary file 1 [file DataSheet1.docx]

Supplementary Material

1. **SUPPLEMENTARY FIGURES**


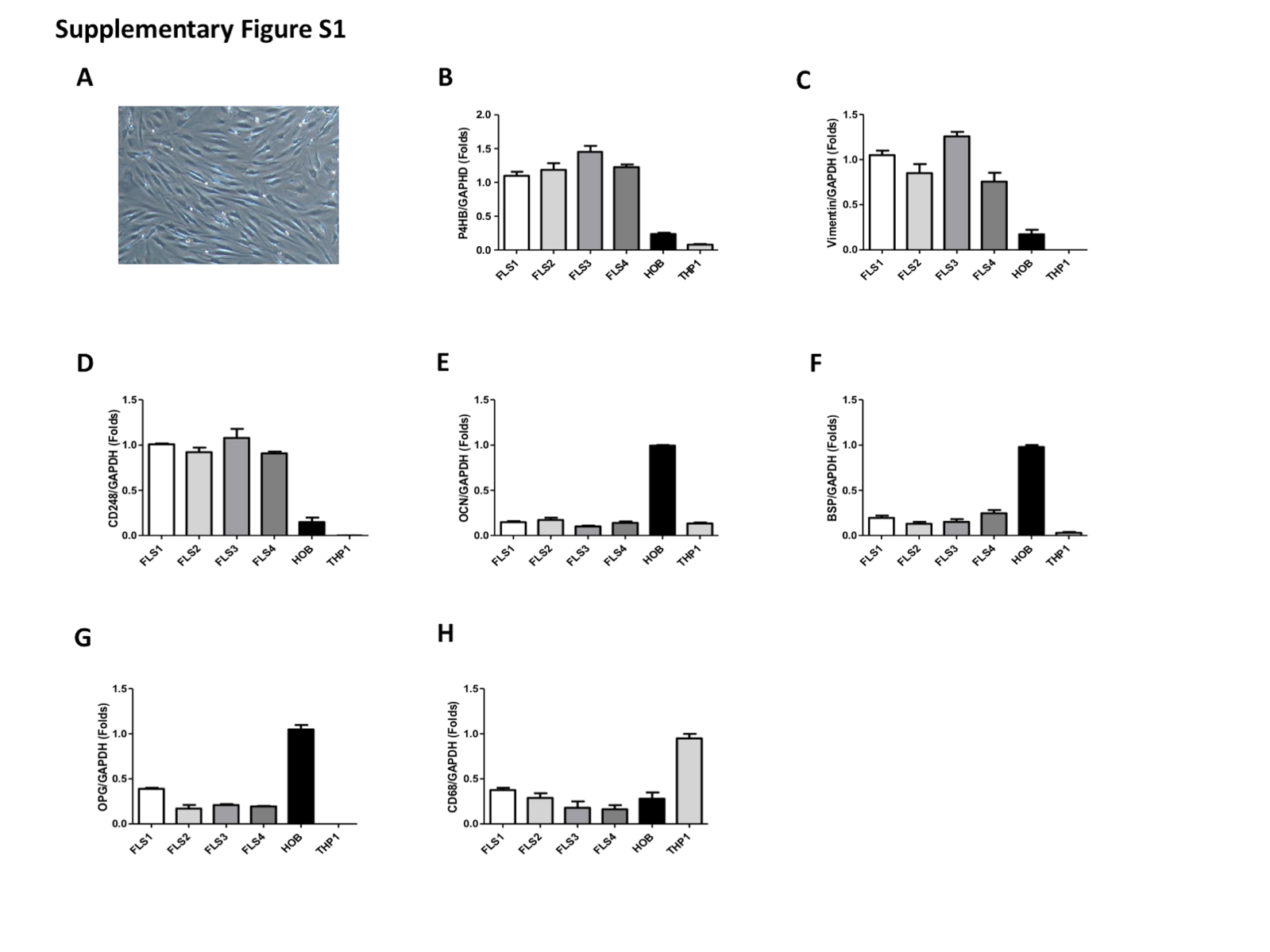


**Figure S1. Characterization of FLS isolated from human synovial tissue.** (A) A confluent monolayer of isolated FLS was grown at passage 2 and observed at 20X magnification. (B-D) mRNA expression levels of fibroblast specific markers such as prolyl 4 hydroxylase (P4HB), vimentin and CD248, (E-G), osteoblast-specific markers like osteocalcin (OCN), bone sialoprotein (BSP), and osteoprogrenin (OPG) and (H) monocyte specific marker CD68 were determined by real-time RT-PCR in FLS at passage 3, primary osteoblasts (HOB) and the monocyte cell line, THP 1. Data are shown as the mean ±SD. Similar results were obtained in three independent experiments.


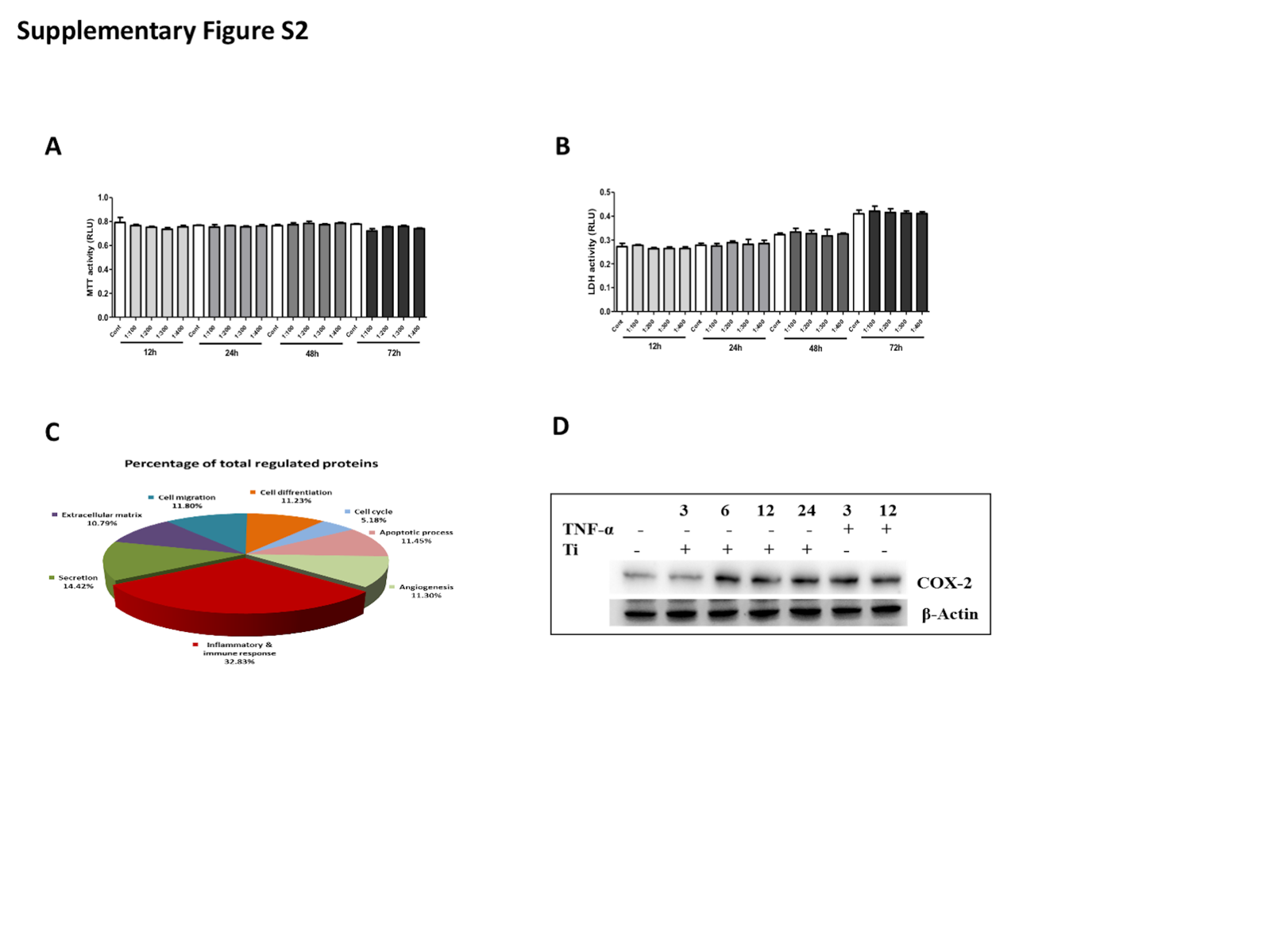


**Figure S2. Effect of Ti particles on FLS.** (A&B) Ratio (FLS to Ti particles) of 1:100-1:400 demonstrated no cell viability (MTT assay) and cytotoxic (LDH assay) effect on FLS until 72 h of treatment. (B) Secretory protein array analysis of Ti CM. Total secreted proteins in the Ti CM are grouped into 9 categories and depicted as a percentage of their ratio to Cont CM. (C) Ti particle treatment-induced COX-2 expression in a time-dependent manner (3, 6, 12, and 24 h). TNF-α was taken as a positive control. Similar results were obtained in three independent experiments.


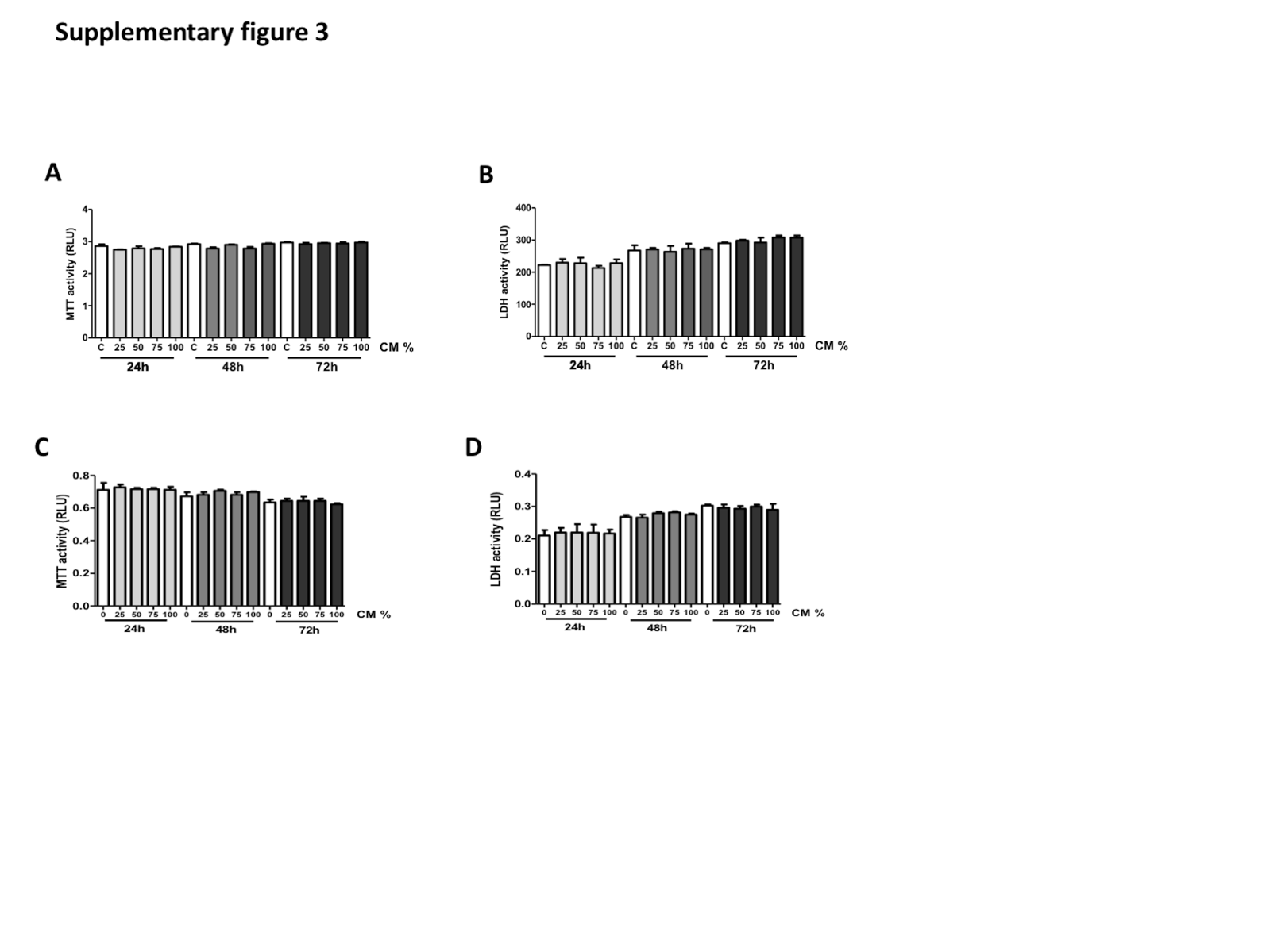


**Figure S3. Effect of Ti particles on cell viability and cytotoxicity in Raw 264.7 cells and osteoblast cell (SaOS-2)** (A&B) Ti CM treated to Raw 264.7 cells and (C&D) SaOS-2 cells at different percentages (25%-100%) demonstrated no cell viability (MTT assay) and cytotoxic (LDH assay) effect till 72 h of treatment. Similar results were obtained in three independent experiments.


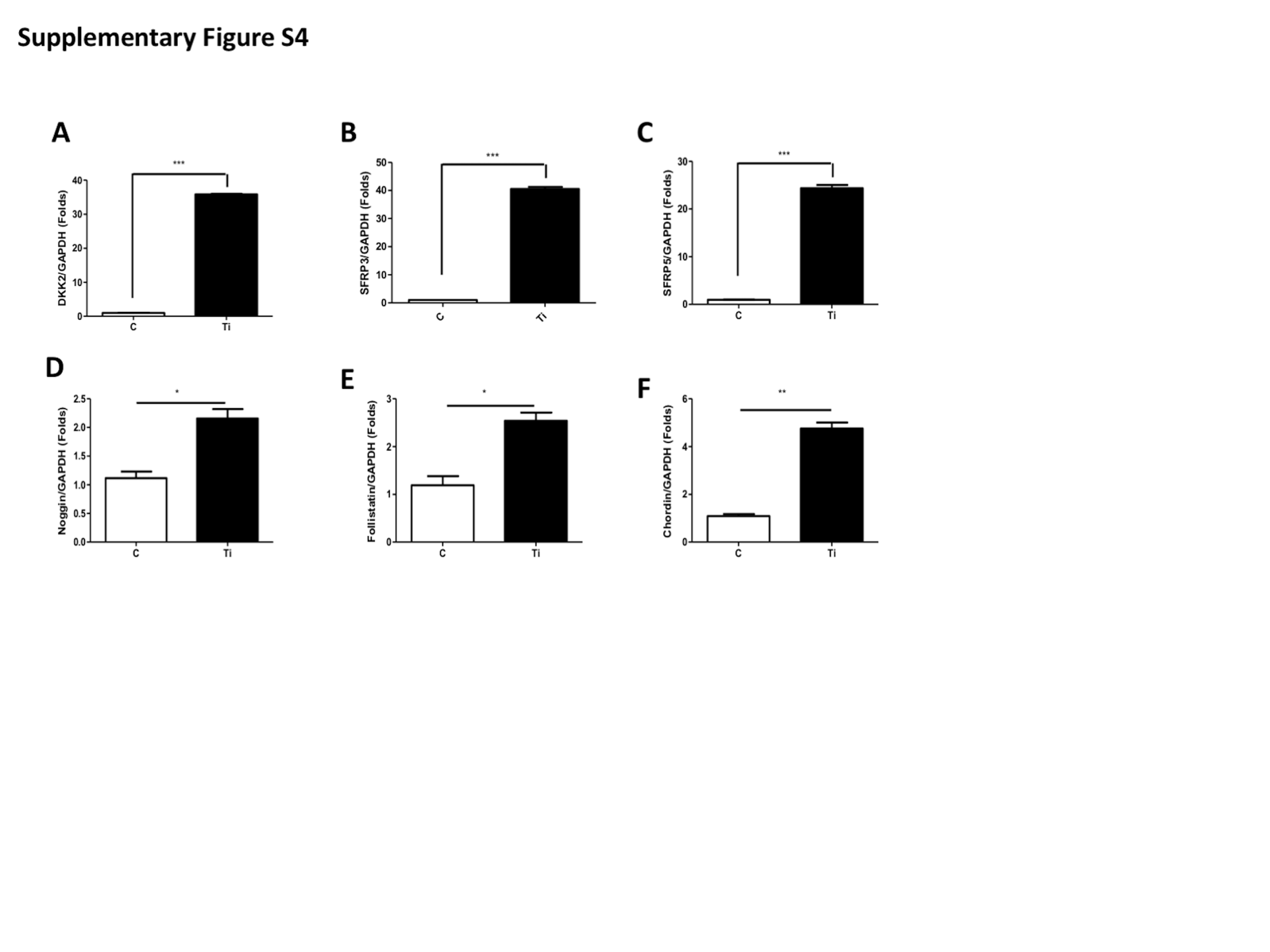


**Figure S4**. **Ti particles induced the expression of WNT and BMP signaling antagonists in FLS.** Treatment of Ti particle to FLS induced mRNA expression of WNT antagonist: (A) DKK2, (B) sFRP3, (C) sFRP5, and BMP signaling antagonist: (D) noggin, (E) chordin, and (F) follistatin. Similar results were obtained in three independent experiments. *P < 0.05, **P < 0.01, ***, P < 0.001 and ns (no significance) compared to the control.
